# The clinical application of SNP-based next-generation sequencing (SNP-NGS) for evaluation of chimerism and microchimerism after HLA-mismatched stem cell microtransplantation

**DOI:** 10.1007/s12185-022-03415-8

**Published:** 2022-07-08

**Authors:** Weiyang Li, Yi Xu, Yufeng Feng, Haixia Zhou, Xiao Ma, Depei Wu, Suning Chen, Aining Sun

**Affiliations:** 1 Jiangsu Institute of Hematology, Key Laboratory of Thrombosis and Hemostasis of Ministry of Health, The First Affiliated Hospital of Soochow University, 188 Shizi Street, Suzhou, 215006 Jiangsu Province People’s Republic of China; 2 National Clinical Research Center for Hematologic Diseases, The First Affiliated Hospital of Soochow University, Suzhou, People’s Republic of China; 3 Institute of Blood and Marrow Transplantation, Collaborative Innovation Center of Hematology, Soochow University, Suzhou, People’s Republic of China; 4 Key Laboratory of Thrombosis and Hemostasis of Ministry of Health, Suzhou, People’s Republic of China

**Keywords:** SNP-NGS, Chimerism, Microchimerism, Microtransplantation

## Abstract

Genetic diagnostic methods for evaluation of chimerism after HSCT, such as STR-PCR and XY-FISH, have limited sensitivity. When donor chimerism is in the micro range (< 1%), deviations in the accuracy of assessment are the most significant disadvantage of these common methods. We developed a highly sensitive method that applies SNPs based on NGS in order to explore the value of donor cell microchimerism in microtransplantation (MST). This improved SNP-NGS approach has higher sensitivity (0.01–0.05%) and only requires a small amount of DNA (8–200 ng). We retrospectively analyzed the clinical data of 48 patients with AML who received HLA-mismatched stem cell MST at our center to assess the impact of microchimerism on clinical prognosis. Patients whose duration of microchimerism was > 10.5 months (median) had a relapse rate of 26.1%, and had better 5-year LFS and OS (73.4% and 82.6%). In contrast, patients whose duration of microchimerism was < 10.5 months had a higher relapse rate (69.6%), and their 5-year LFS and OS were 30.4% and 43.5%. In conclusion, duration of donor chimerism is highly valuable for assessment of survival and prognosis in patients with AML who have received HLA-mismatched stem cell MST, especially the intermediate-risk group.

**Supplementary Information:**

The online version contains supplementary material available at 10.1007/s12185-022-03415-8.

## Introduction

Allogenic hematopoietic stem cell transplantation (allo-HSCT) is the predominant treatment used to cure malignant and non-malignant hematological disorders. Analysis of chimerism following allogeneic HSCT has been a routine method for the assessment of engraftment and early detection of graft failure. It is helpful in guiding risk-adapted immunotherapy for prophylaxis and interventional treatment of graft-versus-host disease (GVHD) or imminent relapse [1, 2]. HLA-mismatched stem cell microtransplantation (MST) is a new therapy protocol of transplantation reported in recent years that could effectively avoid GVHD [3, 4]. A MST treatment method that combines chemotherapy, allo-HSCT, and cellular immunotherapy has shown a good therapeutic effect in adult AML patients [5, 6]. MST treatment improved remission and survival rates, as well as the effect of rapid hematopoietic recovery, without long-lasting allogeneic cell implantation and the major risks of GVHD [7]. MST resulted in persistent or short-term donor microchimerism (< 1%), rather than full or mixed donor chimerism.

Polymerase chain reaction for short tandem repeats (STR-PCR), revealed by conventional agarose-acrylamide gel electrophoresis, is the most commonly used method for analysis of chimerism due to the high informativity and discrimination between donor and recipient. This method has a moderate sensitivity (between 1 and 5%) [8, 9]. Chimerism in sex-mismatched transplantations can be analyzed by using interphase fluorescent in situ hybridization (FISH) with specific probes for the sex chromosomes (XY-FISH). The strengths of this method are its higher sensitivity (0.5–1%) and better quantitative accuracy than what is achievable with most PCR-based assays. Recently, real-time PCR (qPCR), droplet digital PCR (ddPCR) based on STR [10] or next-generation sequencing (NGS) based on single nucleotide polymorphism (SNP) [11, 12] have been evaluated with higher sensitivity and more accurate quantification. These novel methods are suitable for assessment of chimerism in the micro range (< 1%).

The main aim of the present investigation was to develop a custom bioinformatic pipeline for chimerism and microchimerism quantification based on SNP-NGS. We retrieved SNP loci with a frequency of 45–55% in the East Asian population from GenBank and performed linkage disequilibrium analysis to screen 48 SNP loci from 22 different autosomes. It is expected that the resolution of these sites to distinguish donors and recipients is 99.99%. We designed a primer pool for SNPs to achieve the probability of discriminating between donors and recipients. Meanwhile we compared the efficacy of SNP-NGS with that of STR-PCR and XY-FISH for chimerism quantification. In addition, we explored the application of this method in prognosis assessment of MST. To further explore the value of donor cell microchimerism in MST, our center retrospectively analyzed the clinical data of young AML patients who used MST consolidation therapy after the first complete remission and used SNP-NGS to monitor the donor microchimerism post MST and assess the impact of microchimerism on the clinical prognosis of patients.

## Materials and methods

### Patients and sample preparation

Studies were approved by the Human Ethics Committee of the First Affiliated Hospital of Soochow University in accordance with the Declaration of Helsinki protocol. Written informed consent was obtained from all participants. A total of 48 eligible AML patients, 12–59 years of age, were enrolled in this study. Patient clinical characteristics are shown in Table 1. Diagnosis was confirmed according to the World Health Organization (WHO) and French-American-British (FAB) classification criteria. Criteria for eligibility were as follows: patients achieved complete remission (CR) after receiving primary or two remission induction chemotherapies and an Eastern Cooperative Oncology Group (ECOG) performance status of ≤ 2. Risk stratification refers to the 2017 European Leukemia Network (ELN) recommendation. None of these patients had HLA-matched sibling donors or unrelated donors. Before transplantation, donor and recipient HLA-A, -B, -C, -DRB1, and -DQB1 loci were genotyped at high resolution. The median age of donors was 31 years (14–61 years). Genomic DNA was extracted using a Promega DNA extraction kit (Promega, Madison, WI).

**Table 1:** Clinical data for all patients

Characteristics	Low-risk (*n* = 28)	Intermedia-risk (*n* = 14)	High-risk (*n* = 6)
Age, years, median (range)	49 (12–59)	53 (15–59)	56 (48–59)
Gender			
Male	12	5	3
Female	16	9	3
FAB classification			
M0	0	1	0
M1	3	1	1
M2	14	6	4
M4	7	3	0
M5	3	2	1
M6	0	0	0
Undetermined	1	1	0
Disease state			
Past history of MDS	0	0	3
No history of MDS	28	14	3
CR complete remission			
CR1	28	12	5
CR2	0	2	1

Samples of peripheral blood from two healthy volunteer donors (V1–V2) were collected for sensitivity testing of chimerism detection. All peripheral blood samples were separated into mononuclear cells by Ficoll density gradient centrifugation. Mononuclear cell samples from V1 (male) and V2 (female) were paired as “donor” and “recipient,” respectively. Seven artificial chimeric mononuclear cell mixtures, as donor/recipient chimerism, were mixed by counting at seven percentages (10%, 5%, 1%, 0.5%, 0.1%, 0.05%, 0.01%) for each artificial mixture (aCh).

### SNP-NGS

Forty eight single-nucleotide, biallelic, polymorphisms were selected from the Single Nucleotide Polymorphism database (dbSNPs) (http://www.ncbi.nlm.nih.gov/SNP/). The SNP loci of the patients are shown in Fig. S1. 48 primer pairs (Table S1) were designed in the multiplex reaction of amplification donor DNA. A total of 4–6 base sequences were used as label regions for primer design in Primer Pool, such as AGTC, CCAA, GTCT, TAGG, ACAG, TGCT, CTTC, or GAGA. By introducing label regions, it is possible to remove contaminant interference. The multiplex PCR mixture contained 8–200 ng genomic DNA (quantified by Qubit^®^ dsDNA HS Assay Kit), 8 μl PrimerMix (100 nmol/l), and 15 μl high-fidelity PCR Master Mix (Thermo Fisher Scientific, Inc.) in a total volume of 30 μl. The reaction conditions were as follows: 95 ℃ for 3 min (holding stage), 18 cycles of 95 ℃ for 15 s, and 60 ℃ for 4 min (cycling stage). The purification operation was performed using nucleic acid purification kit magnetic beads (Beckman AMPure XP Beads) as target DNA product is bound to magnetic beads. A second round of PCR purification was performed. The SNP-PCR mixture contained the DNA product bound to the beads from the previous step, 1 μl forward universal sequencing primer (F_universal: AATGA TACGG CGACC ACCGA GATCT ACACT CTTTC CCTAC ACGAC GCTCT TCCG), 1 μl barcode sequencing primer (R_index: CAAGC AGAAG ACGGC ATACG AGAT) (10 μmol/l), and 15 μl high-fidelity PCR Master Mix (Thermo Fisher Scientific, Inc.) in a total volume of 30 μl. The reaction conditions were as follows: 95 ℃ for 1 min (holding stage), 6 cycles of 95 ℃ for 15 s, and 60 ℃ for 15 s and 72 ℃ for 15 s (cycling stage). Subsequently, the purification operation was also performed using nucleic acid purification kit magnetic beads (Beckman AMPure XP Beads). Finally, the target PCR product was eluted from the beads and recovered for high-throughput sequencing.

### Genomic analysis

Sequencing results were processed with BWA software (BWA, 2009, PMID19451168) [13]. For the sample amplified by the label primer, the non-sample label sequence is removed by the written Perl script program, and only the sample with the specific label sequence is retained so that sample cross-contamination is better avoided. Only the SNPs labeled informative were used to calculate the chimerism ratio. The sequencing results of chimerism samples were compared with the standard genome to obtain the base sequence of the designated SNP sites. The chimerism ratio is calculated by picking the site where the SNP genotype is inconsistent between the donor and the recipient. The chimerism rate of a single difference SNP is calculated in three scenarios. (1) When the donor and recipient are different homozygotes (such as AA and BB), the calculation formula of the chimerism rate is “Donor% = A/(A + B) × 100%.” (2) When the donor genotype is AA and the recipient genotype is A/B, the calculation formula of the chimerism rate is “Donor% = (A-B)/(A + B) × 100%.” (3) When the donor genotype is A/B and the recipient genotype is AA, the calculation formula of the chimerism rate is “Donor% = 2B/(A + B) × 100%.”

### Mobilization and apheresis of donor peripheral mononuclear cells and treatment design

Donors were subcutaneously injected with 10 μg/kg G-CSF for 5 days to mobilize peripheral hematopoietic stem cells. Apheresis of donor peripheral mononuclear cells was performed using a COBE SPECTRA cell separator (Gambro BCT, Lakewood, CA) and aliquoted into three parts. One of three fresh donor cells was used for the first course, and the remaining parts were cryopreserved in liquid nitrogen for the second and third courses. The median numbers of mononuclear and CD34 + cells infused per course were 2.39 × 108/kg (range, 0.67–6.50 × 10^8^/kg) and 1.32 × 10^6^/kg (range, 0.32–5.12 × 10^6^/kg), respectively.

Remission induction chemotherapy consisted of maintenance 24 h intravenous drip of Ara-C 100 mg/m2 daily for 7 days or idarubicin 12 mg/m2 daily for 3 days. If the patient did not achieve complete remission (CR) after the first course of treatment, a second cycle of the same induction therapy was recommended. Patients who achieved complete remission after one or two cycles of induction therapy were enrolled in this study. These 48 patients who achieved CR1 were assigned to receive three further courses of consolidation chemotherapy consisting of the following: Ara-C 2.0 g/m^2^ per 12 h i.v. on days 1, 2, and 3; idarubicin 10 mg/day i.v. on days 4 and 5; infusion of granulocyte stimulating factor (G-CSF)-mobilized donor consolidation treatment of peripheral blood stem cells (G-PBSCs) on day 7. No preventive treatment of GVHD was given after G-PBSC infusion. The interval between the cycles was 2–2.5 months. G-CSF was given (300 μg/d) when the neutrophil count (ANC) was < 0.5 × 10^9^/L. When the level of hemoglobin was < 70 g/L and platelet count was < 20 × 10^9^/L, red blood cell and platelet transfusion were given, respectively. Antibacterial drugs, antifungal drugs, and antiviral drugs were administered according to institutional guidelines.

### Statistical analysis

SPSS version 19.0 (SPSS, Chicago, IL) software was used for all statistical analyses. Survival data were analyzed by means of log-rank test, and survival curves were generated using the Kaplan–Meier method. Survival curves were generated using the Kaplan–Meier method. The probabilities of OS and LFS were estimated by means of the log-rank test. *P* < 0.05 indicates that the difference is statistically significant.

## Results

### SNP-NGS genotyping performances

A total of 7 artificial chimerism (aCh1-7) were prepared in a fixed detection range (0.01–10.0%). Sequencing results were fully consistent with the known genotype of the donors and recipients. Subsequent to Illumina sequencing and using the custom tool, quantitative data for all SNPs of each artificial chimerism were obtained. The detection results were visually inspected to confirm the presence of donor chimerism or microchimerism.

**Fig. 1:**
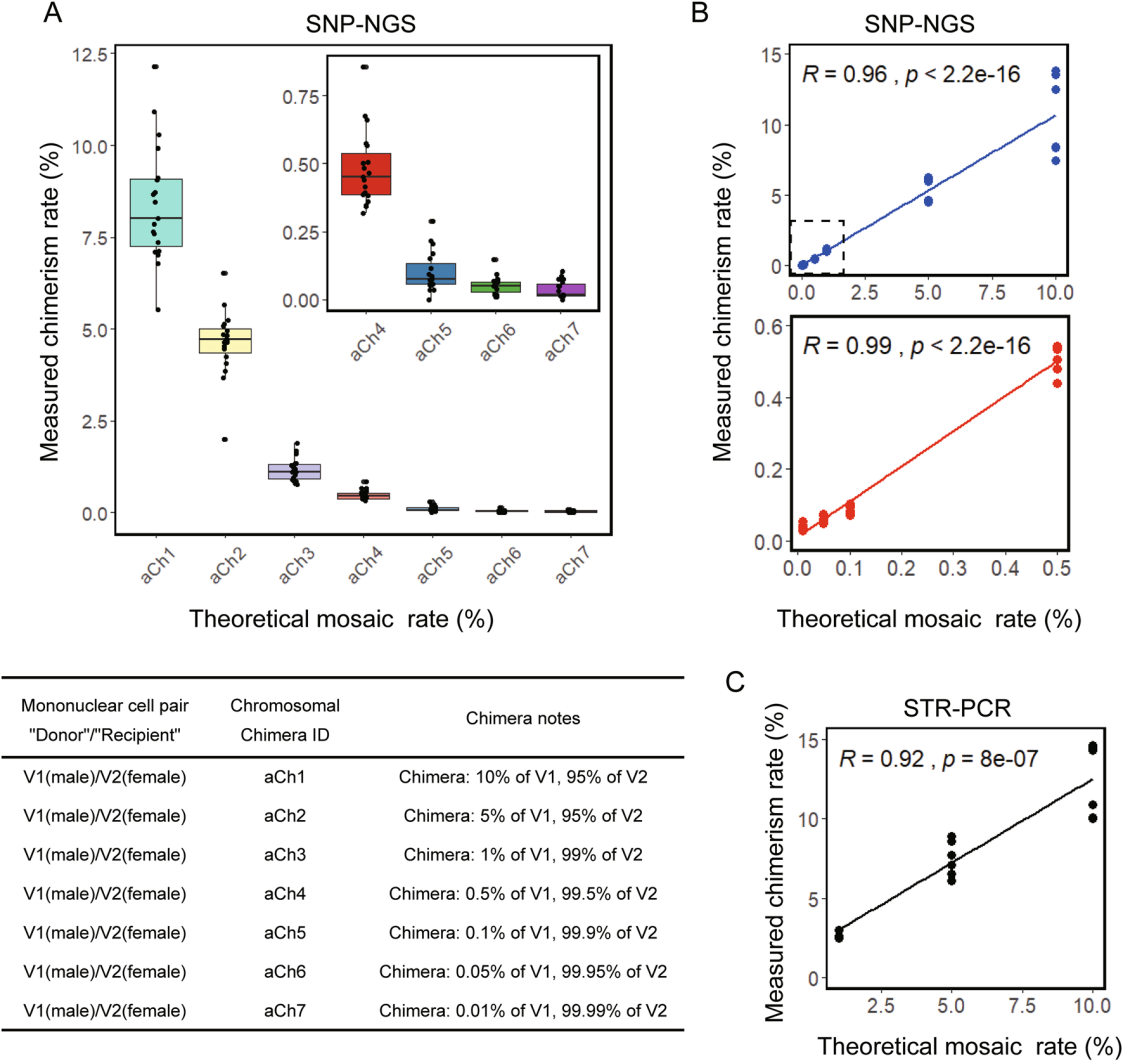
Comparison of the results obtained from chimerism quantification with SNP-NGS and STR-PCR. **A** Box plot of the results obtained by SNP-NGS. Jitter scattered points represent SNP sites. **B** Scatter plot of the results obtained by SNP-NGS. The upper panel shows six sets of data for the series of artificial chimerism (aCh1-aCh7: 10%, 5%, 1%, 0.5%, 0.1%), 0.05%, and 0.01%). The lower panel shows six sets of data for three artificial chimerism (aCh4-aCh7: 0.5%, 0.1%, 0.05% and 0.01%). **C** Scatter plot of the results obtained by multiple STR-PCR

The SNP-NGS analysis of artificial chimerism or microchimerism showed that the limit of detection for a minor cell population is nearly 0.01%. Figure 1A is a box plot of these experimental data. Three separate tests were performed on different chimerism formed in each mixing experiment. The experimental data are provided in supplementary materials (Tables S2-S7). Least-squares analysis of these putative points identified a clear linearity between detection results and the reference values (Fig. 1B).

Multiple STR-PCR was performed using artificial chimerism (aCh1-3) ranging from 1.0 to 10.0% (Fig. 1C). Table S8 indicates the selected informative STRs and their chromosomal locations, which distinguish donors from recipients. The chimeric rate of each STR locus in three replicates is also shown in Table S8. Least-squares analysis identified a clear linearity between STRs and the reference values. XY-FISH was also performed to analyze the chimeric ratio of artificial chimerism (aCh1-6), ranging from 0.05 to 10%. The count results of different chimerism are shown in Table S9. XY-FISH shows a slightly better sensitivity (0.5% when 1,000 cells are scored). Obviously, this method has certain limitations. Comparable results indicated that the improved SNP-NGS technique had better accuracy than STR-PCR and XY-FISH for the quantification of chimerism. Meaningfully, this approach results in an increase in sensitivity of two orders of magnitude compared to the commonly used STR-PCR and XY-FISH methods.

### Detection of donor chimerism and microchimerism

**Fig. 2:**
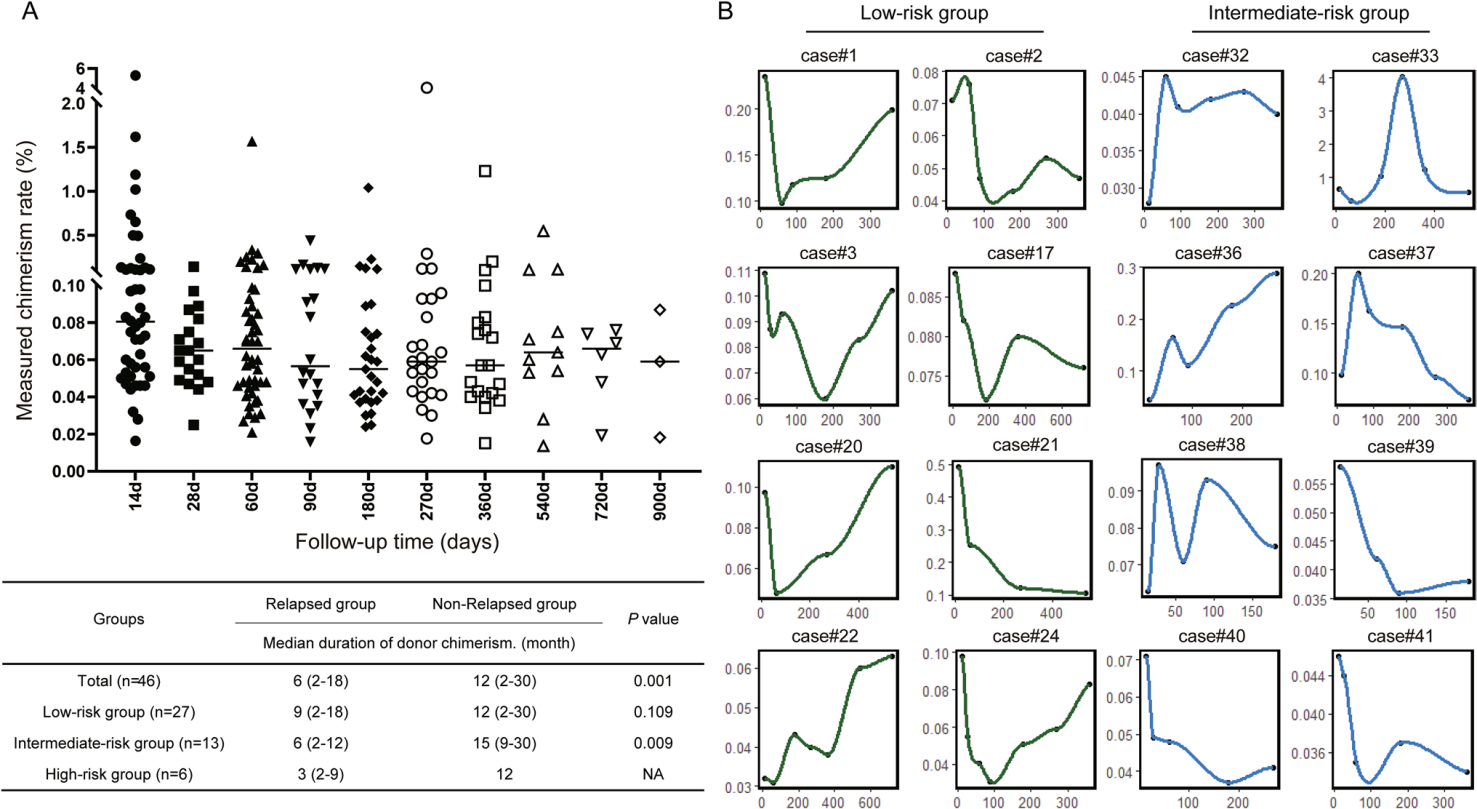
Detection of donor chimerism and microchimerism. **A** The donor cell chimeric dynamics of all patients after microtransplantation. **B** Fitting curve diagram of the results obtained by SNP-NGS for part of low-risk and intermediate-risk patients

All patients were consecutively monitored for donor chimerism or microchimerism using SNP-NGS technology. The donor chimerism or microchimerism were detected at + 14 d, + 28 d, + 2 m, + 3 m, + 6 m, + 9 m, + 12 m, + 18 m, + 24 m, + 30 m, + 36 m after GPBSC infusion. Forty-six patients received long-term chimerism monitoring after completing all consolidation treatments. The remaining two patients only tested for donor chimerism two months after the treatment, one in the low-risk group and another was in the intermediate-risk group. The donor cell chimerism or microchimerism dynamics of all patients are shown in Fig. 2A and dynamic graphs for some patients are shown in Fig. 2B. None of the patients formed a complete or mixed donor chimerism state after microtransplantation. The duration of donor microchimerism was not correlated with the infused mononuclear cells (MNCs) (*R* = 0.121, *P* = 0.144) or the number of CD34^+^ cells (*R* = 0.02, *P* = 0.318) (data not shown). Among 227 samples from 48 patients, 219 samples had microchimerism of less than 1%.

The persistence of chimerism or microchimerism varied from 2 to 30 months. The median was 10.5 months. The median duration of donor chimerism or microchimerism in patients with relapsed disease was 6 months (2–18 months), and the median in patients without relapsed was 12 months (2–30 months). In patients whose persistent time of donor chimerism or microchimerism was < 10.5 months, the relapse rate was 69.6% (16/23). A lower relapse rate (26.1%, 6/23) (*χ*^2^ = 8.712, *P* = 0.003) was found in patients whose persistence of chimerism or microchimerism > 10.5 months. Thirteen patients received long-term monitoring of donor chimerism or microchimerism in intermediate risk group. The median of nonrelapsed and relapsed patients was 15 months (9–30 months) and 6 months (2–12 months), respectively (*P* = 0.009). The 13 cases were divided into two subgroups by a median time of 10.5 months. The relapse rate of the subgroup < 10.5 months was 85.7% (6/7), and lower relapse rates (16.7%, 1/6) (*χ*^2^ = 6.198, *P* = 0.029) were seen in the other subgroup. These data suggested that patients with a long persistence of donor microchimerism have a lower relapse rate.

### LFS and OS

LFS was defined as the time from CR to diagnosis of relapse. OS was defined as the time from assignment to death or to the last observation date. The deadline for follow-up was November 30th, 2019. The median LFS was 50.5 months (3–84 months), and the median OS was 53.5 months (4–84 months). The 5-year LFS and OS rates were 51.6% and 62.5%, respectively (Fig. S2). No patient was lost during the follow-up period. The incidence of relapse in low-risk patients was 35.7% (10/28), of which 3 patients reached CR2 after reinduction therapy, successfully completing myeloablative allo-HSCT to achieve long-term survival. The incidence of relapse in intermediate-risk patients was 57.1% (8/14), of which only one patient achieved long-term survival after treatment after myeloablative allo-HSCT. Five patients in the high-risk group (5/6, 83.3%) experienced relapse, and eventually, all died of the disease. The 5-year LFS rates were 63.3%, 42.9%, and 16.7% in the low-risk, intermediate-risk, and high-risk groups, respectively. The 5-year OS rates were 78.6%, 50.0%, and 16.7% in the low-risk, intermediate-risk, and high-risk groups, respectively (Fig. S2). Pairwise comparisons between low-risk, intermediate-risk, and high-risk groups are statistically significant.

**Fig. 3:**
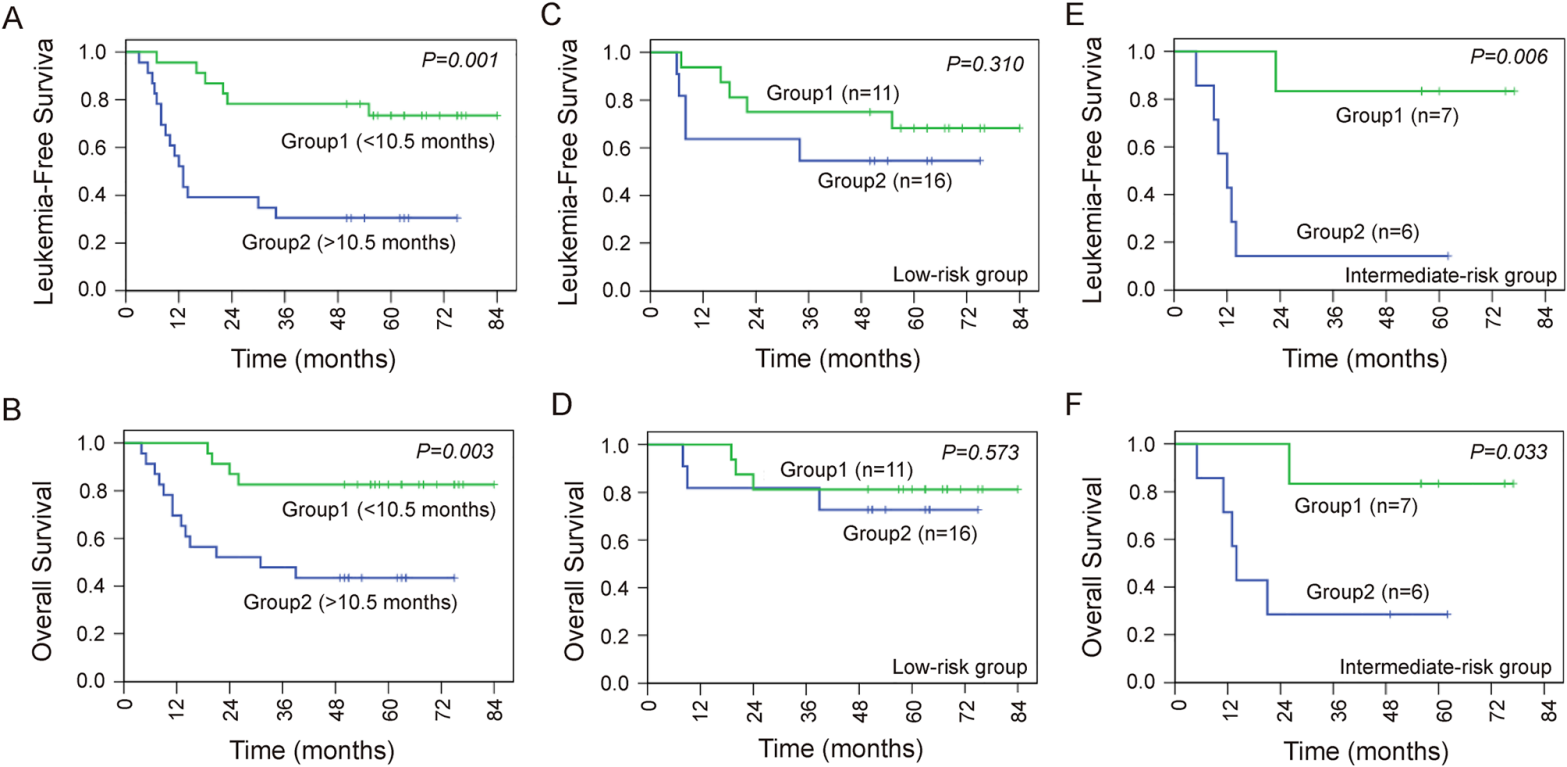
LFS and OS according to the persistence of microchimerism. **A** The 5-year LFS rate was significantly different (*P* = 0.001) between group 1 (73.4%) and group 2 (30.4%). **B** The 5-year OS rate was significantly different (*P* = 0.003) between group 1 (82.6%) and group 2 (43.5%). **C** In the low-risk group, the 5-year LFS rate was not significantly different (*P* = 0.310) between group 1 (*n* = 16, 68.2%) and group 2 (*n* = 11, 54.5%). **D** In the low-risk group, the 5-year OS rate was not significantly different (*P* = 0.573) between group 1 (*n* = 16, 81.3%) and group 2 (*n* = 11, 72.7%). **E** In the intermediate-risk group, the 5-year LFS rate was significantly different (*P* = 0.006) between group 1 (*n* = 6, 83.3%) and group 2 (*n* = 7, 14.3%). **F** In the intermediate-risk group, the 5-year OS rate was significantly different (*P* = 0.033) between group 1 (*n* = 6, 83.3%) and group 2 (*n* = 7, 28.6%)

For further analysis, the 46 patients who received long-term monitoring were divided into 2 groups based on the median of persistent time of donor chimerism or microchimerism (10.5 months). Among them, patients with persistence of chimerism or microchimerism > 10.5 months were group 1, and those with persistence of chimerism or microchimerism < 10.5 months were group 2. The 5-year LFS and OS of group 1 were 73.4% and 82.6%, respectively, and the 5-year LFS and OS of group 2 were 30.4% and 43.5%, respectively (Fig. 3A, B). For 27 patients in the low-risk group, the 5-year LFS and OS of group 1 were 68.2% and 81.3%, respectively, and the 5-year LFS and OS of group 2 were 54.5% and 72.7%, respectively (Fig. 3C, D). For 13 patients in the intermediate-risk group, both the 5-year LFS and OS of group 1 were 83.3%, and the 5-year LFS and OS of group 2 were 14.3% and 28.6%, respectively (Fig. 3E, F). These data suggested that patients with a long persistent time of donor microchimerism have a higher survival rate, especially in the intermediate-risk group.

## Discussion

We improved the detection method to serve the detection of donor microchimerism post MST. In previous study, Tikumphorn Sathirapatya and colleagues [11] used 13 SNPs markers from a TaqMan® Predesigned SNP Genotyping Assay (Applied Biosystems) to provide genotypes from allelic discrimination plot. Their SNP panel could effectively detect 90% of recipient-donor pairs. Jieun Kim and colleagues [12] evaluate the feasibility of SNP-based NGS for detection of microchimerism and the detection limit was defined as ≥ 0.5%. Linjun Chen and colleagues [14] describes the successful clinical application of single-sperm-based SNP haplotyping for the preimplantation genetic diagnosis (PGD) of Osteogenesis imperfecta (OI). They selected 10 of informative SNPs markers via NGS and the minimum test data mentioned in the article is 0.12%. The present investigation focused on the quantification accuracy and described the clinical utility of SNP-NGS for the donor chimerism. First, we used the combination of the 48 SNPs capable of discriminating the “donor” and “recipient” in the artificial chimerism in a preliminary experiment. The experiments were performed using artificial mixtures of male and female samples in different proportions (10%, 5%, 1%, 0.5%, 0.1%, 0.05%, 0.01%). These artificial chimerism were used to test the quantification accuracy of primer pools applied in the SNP-NGS protocol. By introducing label regions, it is possible to remove contaminant interference in the step of amplification of the target area. Particularly for the analysis of large samples, the sensitivity of substance detection could be improved by identifying the label in the sequencing results. Second, compared with the two common methods in clinical, multiplex STR-PCR and XY-FISH, SNP-NGS shows higher sensitivity and quantification accuracy. Moreover, it is not restricted by the gender of the donor and recipient. The results demonstrated that the use of this developed approach increases the sensitivity of the assay to 0.01% and the accuracy to 0.05%. These data highlight the importance of the detection method with high sensitivity and accuracy.

We applied the improved method to serve the detection of donor microchimerism in microtransplantation for AML. We enrolled 48 patients and examined the dynamic changes of their microchimerism using the SNP-NGS method. Remarkably, of the 227 samples, 219 had detectable microchimerism of less than 1% (219/227, 96.48%). These data highlight the importance of the novel detection method with high sensitivity and accuracy. The detection data confirmed that the persistence of donor microchimerism in patients who did not relapse after MST was significantly longer than that of relapsed patients. Moreover, subgroup analysis showed that the persistence of donor microchimerism had a more significant impact on the survival of AML patients in the intermediate-risk group. Patients at intermediate risk with longer persistent time of donor microchimerism had better LFS and OS. In previous reports from our center and the Affiliated Hospital of the Academy of Military Medical Sciences, the hematopoietic recovery time was shorter in the MST group than in the MSD (HLA-matched sibling donor) group. Other advantages of MST are the lower infection rate and lower incidence of GVHD [15, 16]. The exact mechanism for successfully circumventing GVHD after MST is still unclear. Our research also did not find a clear GVHD. In our study, no patient received strong immunosuppressive therapy before MST. This preserved considerable immune function in recipients, important for preventing the occurrence of GVHD.

Our results show that microtransplantation is safe and effective in treating young patients with low- and intermediate-risk AML, and SNP-NGS can accurately detect the microchimerism status of donor cells. Remarkably, the duration of donor microchimerism is of great value for the survival and prognosis assessment of AML patients, especially in the intermediate-risk group. Patients at intermediate risk with longer donor microchimerism have better LFS and OS. Therefore, a well-established universal method is warranted to examine donor-cell microchimerism. Our research mainly focuses on the establishment of methodologies that overcome the limitations of current technologies in sensitivity and accuracy. Improving the accuracy of chimerism detection can help provide important auxiliary means for recurrence monitoring and survival assessment of patients after transplantation.

## Supplementary Information

Below is the link to the electronic supplementary material.

Supplementary file1 (DOCX 62 kb)

Supplementary file2 (PDF 85 kb)

Supplementary file3 (TIF 2722 kb)
