# Supplementary material for: The clinical application of SNP-based next-generation sequencing (SNP-NGS) for evaluation of chimerism and microchimerism after HLA-mismatched stem cell microtransplantation
Source: Int J Hematol. 2022 Jul 8;116(5):723–30. doi: 10.1007/s12185-022-03415-8 (PMC9588463; doi:10.1007/s12185-022-03415-8)
Supplement: Supplementary file 1 — Supplementary file1 (DOCX 62 kb) [file 12185_2022_3415_MOESM1_ESM.docx]

**Supplemental Material**

Fig.S1 SNPs positions in all patients.

Fig.S2 LFS and OS rates according to the risk stratification.

(A) LFS in 48 microtransplant patients. The 5-year LFS rate was 51.6% (n=48).

(B) OS in 48 microtransplant patients. The 5-year OS rate was 62.5% (n=48).

(C) The 5-year LFS in the low-risk group, intermediate-risk group, and high-risk group. The 5-year LFS probability of each group was as follows: low-risk group, 63.3%, n=28; intermediate-risk group, 42.9%, n=14; and high-risk group, 16.7%, n=6. The 5-year LFS rate was significantly different between the low-risk and high-risk groups (*P*< 0.05).

(D) The 5-year OS in the low-risk group, intermediate-risk group, and high-risk group. The 5-year OS probability of each group was as follows: low-risk group, 78.6%, n=28; intermediate-risk group, 50.0%, n=14; and high-risk group, 16.7%, n=6. The 5-year OS rate was significantly different between the low-risk and intermediate-risk groups (*P*< 0.05), and the 5-year OS was also significantly different between the low-risk and high-risk groups (*P*< 0.05).

Table S1. List of dbSNP ID and sequences of primers for SNP-NGS.

Table S2-S7. Chimeric ratio detected in SNPs positions for each artificial chimera.

Table S8. The informative STR loci and the results of multiple STR-PCR.

Table S9. XY-FISH results for each artificial mixture.

Table S1. List of all SNPs and sequences of primers for SNP-NGS.

| No. | dbSNP ID | Chromosome location | Nucleotide  Variation |  | Primers Pool |
| --- | --- | --- | --- | --- | --- |
| 1 | rs10140583 | 14 | A>G | F | CCTACACGACGCTCTTCCGATCT-label-GCTATACAATGTTTGTGTTTCAAGCTAAGTG |
|  |  |  |  | R | gtTCCTTGGCACCCGAGAATTCCA-label-TTTTTCCTTCAATAATAAATCAGCCTTAGCTTA |
| 2 | rs10770083 | 11 | A>G | F | CCTACACGACGCTCTTCCGATCT-label-AAAAACCCATCCCTGGAACTAATAAG |
|  |  |  |  | R | gtTCCTTGGCACCCGAGAATTCCA-label-ATGGTATTATATTTTTGCCTCAAATTCCACTTA |
| 3 | rs10913037 | 1 | A>G | F | CCTACACGACGCTCTTCCGATCT-label-CCCTTTGAGAGCCTATGTGTCA |
|  |  |  |  | R | gtTCCTTGGCACCCGAGAATTCCA-label-TCCAAAATGTGTTTTCTTTGTCACCAAAA |
| 4 | rs11076022 | 16 | A>G | F | CCTACACGACGCTCTTCCGATCT-label-AGAACCTTGTCAGCCAAGTTCTT |
|  |  |  |  | R | gtTCCTTGGCACCCGAGAATTCCA-label-CCACTCAGGAATCCAAAGATTTGGAAA |
| 5 | rs11633588 | 15 | A>G | F | CCTACACGACGCTCTTCCGATCT-label-AGATAGTAACAGTGTTTTAAGCACTTTGGA |
|  |  |  |  | R | gtTCCTTGGCACCCGAGAATTCCA-label-CATGGGCAAGTGACCTCTGTAG |
| 6 | rs11686988 | 2 | C>T | F | CCTACACGACGCTCTTCCGATCT-label-AGCAGGTACTTTGTCAGAAATGCA |
|  |  |  |  | R | gtTCCTTGGCACCCGAGAATTCCA-label-TAGCTCAGTGGTTTCAAACTTTATTGAGT |
| 7 | rs11751424 | 6 | A>G | F | CCTACACGACGCTCTTCCGATCT-label-GCCTCATAAGATAGATAATGCTGTTACCA |
|  |  |  |  | R | gtTCCTTGGCACCCGAGAATTCCA-label-TTTCTTTAAGAGGGTCTCTCTCATCCA |
| 8 | rs12455311 | 18 | A>G | F | CCTACACGACGCTCTTCCGATCT-label-AGAGGAAATCTGTATGTGTAGA |
|  |  |  |  | R | gtTCCTTGGCACCCGAGAATTCCA-label-GCTGGGAACAATATACTATTTGCT |
| 9 | rs1290018 | 11 | C>T | F | CCTACACGACGCTCTTCCGATCT-label-TTTTGGTCCAGGTAACTTGGATTCAT |
|  |  |  |  | R | gtTCCTTGGCACCCGAGAATTCCA-label-CAAAGGACATACTTCACATTTCTTTAGCAC |
| 10 | rs1327362 | 9 | T>C | F | CCTACACGACGCTCTTCCGATCT-label-CACGTCATGAGAAACTACTTGCCT |
|  |  |  |  | R | gtTCCTTGGCACCCGAGAATTCCA-label-GTAGAAGAGACAATAAATGGGATATGTGGT |
| 11 | rs1391057 | 3 | A>G | F | gtTCCTTGGCACCCGAGAATTCCA-label-ATAAAAGTCTTGCAGGGACATCCTTT |
|  |  |  |  | R | gtTCCTTGGCACCCGAGAATTCCA-label-GCAGATTCACAAAGAATCTTAACTAGGAGA |
| 12 | rs1473717 | 20 | C>G | F | CCTACACGACGCTCTTCCGATCT-label-GCATATGCATACATGAGTTGCAGATG |
|  |  |  |  | R | gtTCCTTGGCACCCGAGAATTCCA-label-ATGCCAAAAGTGGAAGCCAGAAGT |

Continue (Table S1)

| No. | dbSNP ID | Chromosome location | Nucleotide  Variation |  | Primers Pool |
| --- | --- | --- | --- | --- | --- |
| 13 | rs1507072 | 17 | T>C | F | CCTACACGACGCTCTTCCGATCT-label-CCTCTTTGAAAGGCACTGAGAACT |
|  |  |  |  | R | gtTCCTTGGCACCCGAGAATTCCA-label-GCAACAAGAGCTAAATGAATCTCATTCTTA |
| 14 | rs1511491 | 15 | T>C | F | CCTACACGACGCTCTTCCGATCT-label-CCATGTAGCACCCAGTGAAAGA |
|  |  |  |  | R | gtTCCTTGGCACCCGAGAATTCCA-label-CTTCACGGCTGATAAAGAAGACAC |
| 15 | rs1786908 | 10 | G>T | F | CCTACACGACGCTCTTCCGATCT-label-CTTAGAGACCTGTGATTACACACATGT |
|  |  |  |  | R | gtTCCTTGGCACCCGAGAATTCCA-label-GGAATCTGAAAATCCCTCAACTGCTTA |
| 16 | rs1989161 | 7 | C>T | F | CCTACACGACGCTCTTCCGATCT-label-TCTTTCTCAGATCTTGTTACCAATTTTCCA |
|  |  |  |  | R | gtTCCTTGGCACCCGAGAATTCCA-label-GATATTCAGTTTGTTTGGAAGGAAGGATG |
| 17 | rs2211012 | 1 | G>T | F | CCTACACGACGCTCTTCCGATCT-label-GGAAAGTCAATCAAAGCCAAATGTAGT |
|  |  |  |  | R | gtTCCTTGGCACCCGAGAATTCCA-label-GGTCAGGGATGGGACCATTTAAAAA |
| 18 | rs2229611 | 17 | T>C | F | CCTACACGACGCTCTTCCGATCT-label-CCGCACAAGAAGTCGTTGTAAG |
|  |  |  |  | R | gtTCCTTGGCACCCGAGAATTCCA-label-CAGAATGGATGGCATGTCACTTG |
| 19 | rs2237117 | 6 | C>G | F | CCTACACGACGCTCTTCCGATCT-label-GGGCCAGGCCTAGGTTTAGTA |
|  |  |  |  | R | gtTCCTTGGCACCCGAGAATTCCA-label-GCCCAGAGGGTACTGAAACAC |
| 20 | rs2348478 | 2 | A>G | F | CCTACACGACGCTCTTCCGATCT-label-GAGGAAGGCGAGATGCTGAAT |
|  |  |  |  | R | gtTCCTTGGCACCCGAGAATTCCA-label-GCAATGCTCTGGCTTGGTACA |
| 21 | rs2469524 | 8 | G>A | F | CCTACACGACGCTCTTCCGATCT-label-CTTTTCAATTTGCCAGCTTCACAAC |
|  |  |  |  | R | gtTCCTTGGCACCCGAGAATTCCA-label-AGAACCTGGTAATTTGTTTTGATTATGTGC |
| 22 | rs2749612 | 10 | G>A | F | CCTACACGACGCTCTTCCGATCT-label-CTCACAATTGCTTTTCTCATCACACGAG |
|  |  |  |  | R | gtTCCTTGGCACCCGAGAATTCCA-label-CCCATTCCAAACCCTCCACAA |
| 23 | rs335746 | 19 | C>A | F | CCTACACGACGCTCTTCCGATCT-label-ACCTGGGAAACTAGCCTTCTGA |
|  |  |  |  | R | gtTCCTTGGCACCCGAGAATTCCA-label-ACTGAGGTACTTTTCAGCCATTTGG |
| 24 | rs34150396 | 8 | A>G | F | CCTACACGACGCTCTTCCGATCT-label-CGCCGTCCTCAAGGTCAATA |
|  |  |  |  | R | gtTCCTTGGCACCCGAGAATTCCA-label-GGAGTTTGCACGGCTACAGT |

Continue (Table S1)

| No. | dbSNP ID | Chromosome location | Nucleotide  Variation |  | Primers Pool |
| --- | --- | --- | --- | --- | --- |
| 25 | rs34471356 | 15 | A>T | F | CCTACACGACGCTCTTCCGATCT-label-CTCAGGTCTAGTCCAGACTCCTT |
|  |  |  |  | R | gtTCCTTGGCACCCGAGAATTCCA-label-GAGCTCCAAGGAATATATAGGCATTACAA |
| 26 | rs34844119 | 3 | G>A | F | CCTACACGACGCTCTTCCGATCT-label-CATTTTCCTCCAAGATATCAGGACCTT |
|  |  |  |  | R | gtTCCTTGGCACCCGAGAATTCCA-label-TTTCAAACATAACCAGTTTGCATGTTGA |
| 27 | rs35475992 | 21 | A>C | F | CCTACACGACGCTCTTCCGATCT-label-GAGAATCAATCCCACCCACTTCT |
|  |  |  |  | R | gtTCCTTGGCACCCGAGAATTCCA-label-GCAACATGCTTTCAAATTCAGGTGAA |
| 28 | rs36064103 | 4 | G>A | F | CCTACACGACGCTCTTCCGATCT-label-GCTGAGCGCCTGGATACAATAA |
|  |  |  |  | R | gtTCCTTGGCACCCGAGAATTCCA-label-GCAAGTTCCAGAATCCGAAGTCT |
| 29 | rs3861308 | 18 | C>T | F | CCTACACGACGCTCTTCCGATCT-label-AAAGGTGCAGATTCTTTTGACATAGAGA |
|  |  |  |  | R | gtTCCTTGGCACCCGAGAATTCCA-label-CAGATCTTTCTGTGCAGCCAGATA |
| 30 | rs4333997 | 11 | T>C | F | CCTACACGACGCTCTTCCGATCT-label-CCCAGTCTGTTCCTCTGCATTA |
|  |  |  |  | R | gtTCCTTGGCACCCGAGAATTCCA-label-GGAAAAGAGAATTCCCTCTTGCACT |
| 31 | rs4350445 | 12 | G>T | F | CCTACACGACGCTCTTCCGATCT-label-CGGCCTTCCTCCTTTTTATTCTT |
|  |  |  |  | R | gtTCCTTGGCACCCGAGAATTCCA-label-CAACAGTAGAGAGAATGGACTGGAG |
| 32 | rs468696 | 21 | C>T | F | CCTACACGACGCTCTTCCGATCT-label-GTGAAATTTCTGTAAGTGCCTGTTCA |
|  |  |  |  | R | gtTCCTTGGCACCCGAGAATTCCA-label-GCAAAATCTCCTTGGGTTGAGTAGAG |
| 33 | rs4698189 | 4 | C>T | F | CCTACACGACGCTCTTCCGATCT-label-CAGTCTAGTCTCCTTTAGATTTACAGCTTT |
|  |  |  |  | R | gtTCCTTGGCACCCGAGAATTCCA-label-ACAGATGGATAGTGAATGGCAGAAAG |
| 34 | rs4820107 | 22 | G>T | F | CCTACACGACGCTCTTCCGATCT-label-TTGGCACGGCCACACAAAGTAA |
|  |  |  |  | R | gtTCCTTGGCACCCGAGAATTCCA-label-CCAGCAATCAGAGAGGAGATCAGA |
| 35 | rs57614941 | 12 | G>A | F | CCTACACGACGCTCTTCCGATCT-label-TCTAGAGAAGCCTCAGGCAGTT |
|  |  |  |  | R | gtTCCTTGGCACCCGAGAATTCCA-label-TTCACACAAGATCTGTTTGTTTAAAAGCAT |
| 36 | rs5768331 | 22 | G>A | F | CCTACACGACGCTCTTCCGATCT-label-CTTGTCCCACGTGAGGCAATA |
|  |  |  |  | R | gtTCCTTGGCACCCGAGAATTCCA-label-CCCATAGGCACTCCCTTGAGA |

Continue (Table S1)

| No. | dbSNP ID | Chromosome location | Nucleotide  Variation |  | Primers Pool |
| --- | --- | --- | --- | --- | --- |
| 37 | rs58385530 | 14 | C>T | F | CCTACACGACGCTCTTCCGATCT-label-GCTTTTTAAGTAAATTCCTTCCTATGTGCA |
|  |  |  |  | R | gtTCCTTGGCACCCGAGAATTCCA-label-AAGGGAGGTTTTTAACTTGACTTAGCTAG |
| 38 | rs6034343 | 20 | C>T | F | CCTACACGACGCTCTTCCGATCT-label-GGTGGAAGGCATTTCTCTCCAT |
|  |  |  |  | R | gtTCCTTGGCACCCGAGAATTCCA-label-TTCTTTTGTGGATCTATCAACAGCTCAA |
| 39 | rs635071 | 13 | A>G | F | CCTACACGACGCTCTTCCGATCT-label-AGGGAGTAAGGGCTGAAAAACG |
|  |  |  |  | R | gtTCCTTGGCACCCGAGAATTCCA-label-GGATACATGTGAAGTTTGTTACGTGGATA |
| 40 | rs6472711 | 8 | A>C | F | CCTACACGACGCTCTTCCGATCT-label-TATTCTTCCAATAATACCAGGCACCAAAA |
|  |  |  |  | R | gtTCCTTGGCACCCGAGAATTCCA-label-GCAAAGCAGCAGAGTTCAATCTC |
| 41 | rs6869226 | 5 | T>G | F | CCTACACGACGCTCTTCCGATCT-label-TCAGTTTTCCTAACTTTGCATGACCA |
|  |  |  |  | R | gtTCCTTGGCACCCGAGAATTCCA-label-GCTGCTAAATGATGTTTACTAGTTTTCCAA |
| 42 | rs75336684 | 12 | A>G | F | CCTACACGACGCTCTTCCGATCT-label-GATGTAGAAGGAACCCACAGACA |
|  |  |  |  | R | gtTCCTTGGCACCCGAGAATTCCA-label-CAAAACCCTCCTCAGCCTTCAA |
| 43 | rs7667671 | 4 | T>C | F | CCTACACGACGCTCTTCCGATCT-label-CATTTCCTTTAGACCTTTAAGATGAAATTGTT |
|  |  |  |  | R | gtTCCTTGGCACCCGAGAATTCCA-label-AAGGCTTGAAATAATAACTCTATGTTCAACTCA |
| 44 | rs7953166 | 12 | A>G | F | CCTACACGACGCTCTTCCGATCT-label-CAGCTGAGCTGTCACCTCATTT |
|  |  |  |  | R | gtTCCTTGGCACCCGAGAATTCCA-label-GCAGTCACTGTCCTTTTGACATGAA |
| 45 | rs842293 | 9 | G>T | F | CCTACACGACGCTCTTCCGATCT-label-GCTACAAATGATAACCAAGCAATATTGGT |
|  |  |  |  | R | gtTCCTTGGCACCCGAGAATTCCA-label-CTATCCTGCAATTCAGTCAAGTCCT |
| 46 | rs9457807 | 6 | A>G | F | CCTACACGACGCTCTTCCGATCT-label-GCCATGTGGATGTAGGGTTCAT |
|  |  |  |  | R | gtTCCTTGGCACCCGAGAATTCCA-label-GAGCAAGGACTCTGCCTTAGTTAC |
| 47 | rs9564669 | 13 | C>T | F | CCTACACGACGCTCTTCCGATCT-label-TCTCCTCAGTCTACACAATTTCCCT |
|  |  |  |  | R | gtTCCTTGGCACCCGAGAATTCCA-label-GGGAGTGGGACGTATTTTAGGC |
| 48 | rs9862757 | 3 | C>T | F | CCTACACGACGCTCTTCCGATCT-label-GGCACCAAGATTTTTCAGCATTAGAG |
|  |  |  |  | R | gtTCCTTGGCACCCGAGAATTCCA-label-GCTACTGTGTTATCACTGCTTCCA |

Table S2. Chimeric ratio detected in SNPs positions for each artificial chimera (the first test results of the first mixing experiment).

| Site | Receptor Genotypes | Donor Genotypes | Sample 1  (aCh1) | Sample 2  (aCh2) | Sample 3  (aCh3) | Sample 4  (aCh4) | Sample 5  (aCh5) | Sample 6  (aCh6) | Sample 7  (aCh7) |
| --- | --- | --- | --- | --- | --- | --- | --- | --- | --- |
| rs10140583 | G/G | A/A | 0.086772 | 0.044612 | 0.008350 | 0.004664 | 0.001072 | 0.000379 | 0.000338 |
| rs10770083 | A/A | G/G | 0.102994 | 0.050822 | 0.010554 | 0.004135 | 0.000725 | 0.000310 | 0.000085 |
| rs11076022 | A/A | G/G | 0.109209 | 0.046381 | 0.011072 | 0.004820 | 0.000867 | 0.000182 | 0.000153 |
| rs11686988 | T/T | C/C | 0.084572 | 0.065342 | 0.009623 | 0.005750 | 0.000890 | 0.000301 | 0.000102 |
| rs1290018 | C/C | C/T | 0.087258 | 0.047239 | 0.007316 | 0.004390 | 0.000765 | 0.000349 | 0.000124 |
| rs1507072 | T/T | C/C | 0.076484 | 0.048489 | 0.011372 | 0.003411 | 0.001043 | 0.000430 | 0.000000 |
| rs2211012 | G/G | T/T | 0.078586 | 0.056586 | 0.009498 | 0.003812 | 0.001034 | 0.000000 | 0.000000 |
| rs2348478 | G/G | A/G | 0.099279 | 0.049574 | 0.012096 | 0.006623 | 0.000000 | 0.001179 | 0.000280 |
| rs2469524 | G/G | G/A | 0.071189 | 0.042441 | 0.009509 | 0.003924 | 0.002631 | 0.001420 | 0.001089 |
| rs2749612 | A/A | G/A | 0.091034 | 0.038641 | 0.008965 | 0.006734 | 0.000850 | 0.000691 | 0.000252 |
| rs335746 | C/C | A/A | 0.080029 | 0.047144 | 0.009790 | 0.005028 | 0.000342 | 0.000366 | 0.000343 |
| rs34471356 | A/A | A/T | 0.067752 | 0.051256 | 0.010336 | 0.005042 | 0.000354 | 0.000327 | 0.000353 |
| rs4333997 | C/C | T/C | 0.076083 | 0.048114 | 0.006770 | 0.004517 | 0.001194 | 0.000744 | 0.000336 |
| rs4350445 | G/G | G/T | 0.055319 | 0.019900 | 0.008271 | 0.003460 | 0.000589 | 0.002274 | 0.000579 |
| rs468696 | C/C | C/T | 0.070073 | 0.036617 | 0.006973 | 0.005684 | 0.000445 | 0.000335 | 0.000000 |
| rs6034343 | C/C | T/T | 0.070944 | 0.040544 | 0.019596 | 0.003182 | 0.000525 | 0.000357 | 0.000242 |
| rs7953166 | A/A | G/G | 0.090494 | 0.052317 | 0.004467 | 0.003878 | 0.000315 | 0.000193 | 0.000290 |
| rs842293 | G/G | G/T | 0.121445 | 0.046512 | 0.010785 | 0.008570 | 0.000961 | 0.000742 | 0.000177 |
| rs9564669 | C/C | C/T | 0.073670 | 0.045353 | 0.010769 | 0.003602 | 0.001150 | 0.000633 | 0.000404 |
| Detection ratio (Average) | - | - | 0.083852 | 0.046204 | 0.009795 | 0.004801 | 0.000829 | 0.000590 | 0.000271 |
| Result judgement | - | - | TURE | TURE | TURE | TURE | TURE | TURE | BIAS |

Table S3. Chimeric ratio detected in SNPs positions for each artificial chimera (the second test results of the first mixing experiment).

| Site | Receptor Genotypes | Donor Genotypes | Sample 1  (aCh1) | Sample 2  (aCh2) | Sample 3  (aCh3) | Sample 4  (aCh4) | Sample 5  (aCh5) | Sample 6  (aCh6) | Sample 7  (aCh7) |
| --- | --- | --- | --- | --- | --- | --- | --- | --- | --- |
| rs10140583 | G/G | A/A | 0.055755 | 0.042697 | 0.010657 | 0.005353 | 0.000676 | 0.000708 | 0.000657 |
| rs10770083 | A/A | G/G | 0.084121 | 0.049459 | 0.009277 | 0.005066 | 0.000551 | 0.000385 | 0.000139 |
| rs11076022 | A/A | G/G | 0.089744 | 0.058476 | 0.009198 | 0.005510 | 0.000773 | 0.000146 | 0.000192 |
| rs11686988 | T/T | C/C | 0.115894 | 0.050352 | 0.009002 | 0.003778 | 0.000852 | 0.000516 | 0.000189 |
| rs1290018 | C/C | C/T | 0.060325 | 0.035114 | 0.009207 | 0.004962 | 0.000849 | 0.000205 | 0.000152 |
| rs1507072 | T/T | C/C | 0.077419 | 0.054496 | 0.012941 | 0.003146 | 0.000484 | 0.000627 | 0.000136 |
| rs2211012 | G/G | T/T | 0.077185 | 0.055916 | 0.008635 | 0.004596 | 0.000610 | 0.000273 | 0.000129 |
| rs2348478 | G/G | A/G | 0.051282 | 0.047874 | 0.007282 | 0.005461 | 0.001480 | 0.000831 | 0.000192 |
| rs2469524 | G/G | G/A | 0.089127 | 0.041472 | 0.008330 | 0.005465 | 0.001548 | 0.000838 | 0.001028 |
| rs2749612 | A/A | G/A | 0.077954 | 0.051502 | 0.005880 | 0.004764 | 0.001107 | 0.000509 | 0.000739 |
| rs335746 | C/C | A/A | 0.093117 | 0.044608 | 0.008789 | 0.003716 | 0.000421 | 0.000242 | 0.000159 |
| rs34471356 | A/A | A/T | 0.080169 | 0.042163 | 0.007902 | 0.003558 | 0.000658 | 0.000333 | 0.000489 |
| rs4333997 | C/C | T/C | 0.080935 | 0.040474 | 0.008221 | 0.005842 | 0.000785 | 0.000753 | 0.000854 |
| rs4350445 | G/G | G/T | 0.090909 | 0.035260 | 0.004331 | 0.002569 | 0.002944 | 0.000351 | 0.000000 |
| rs468696 | C/C | C/T | 0.088205 | 0.045825 | 0.006829 | 0.003739 | 0.000596 | 0.000529 | 0.000492 |
| rs6034343 | C/C | T/T | 0.072222 | 0.035177 | 0.006889 | 0.002899 | 0.000615 | 0.000297 | 0.000337 |
| rs7953166 | A/A | G/G | 0.119658 | 0.040030 | 0.007750 | 0.004167 | 0.000760 | 0.000238 | 0.000135 |
| rs842293 | G/G | G/T | 0.130641 | 0.042316 | 0.012322 | 0.005194 | 0.001007 | 0.000703 | 0.000751 |
| rs9564669 | C/C | C/T | 0.071799 | 0.038850 | 0.010807 | 0.003427 | 0.000902 | 0.000307 | 0.000504 |
| Detection ratio (Average) | - | - | 0.084551 | 0.044845 | 0.008645 | 0.004380 | 0.000927 | 0.000463 | 0.000383 |
| Result judgement | - | - | TURE | TURE | TURE | TURE | TURE | TURE | BIAS |
|  |  |  |  |  |  |  |  |  |  |

Table S4. Chimeric ratio detected in SNPs positions for each artificial chimera (the third test results of the first mixing experiment).

| Site | Receptor Genotypes | Donor Genotypes | Sample 1  (aCh1) | Sample 2  (aCh2) | Sample 3  (aCh3) | Sample 4  (aCh4) | Sample 5  (aCh5) | Sample 6  (aCh6) | Sample 7  (aCh7) |
| --- | --- | --- | --- | --- | --- | --- | --- | --- | --- |
| rs10140583 | G/G | A/A | 0.081882 | 0.051331 | 0.012060 | 0.004840 | 0.000578 | 0.000663 | 0.000416 |
| rs10770083 | A/A | G/G | 0.082500 | 0.054111 | 0.009342 | 0.004394 | 0.000533 | 0.000525 | 0.000119 |
| rs11076022 | A/A | G/G | 0.082324 | 0.046217 | 0.008840 | 0.005022 | 0.000949 | 0.000107 | 0.000153 |
| rs11686988 | T/T | C/C | 0.103306 | 0.055938 | 0.008514 | 0.004584 | 0.000348 | 0.000205 | 0.000141 |
| rs1290018 | C/C | C/T | 0.092937 | 0.046474 | 0.008644 | 0.005109 | 0.000545 | 0.000658 | 0.000108 |
| rs1507072 | T/T | C/C | 0.033557 | 0.042809 | 0.007646 | 0.004919 | 0.000000 | 0.000930 | 0.000000 |
| rs2211012 | G/G | T/T | 0.088099 | 0.053093 | 0.008078 | 0.004748 | 0.000833 | 0.000388 | 0.000169 |
| rs2348478 | G/G | A/G | 0.083636 | 0.046568 | 0.008523 | 0.007343 | 0.001512 | 0.000637 | 0.001449 |
| rs2469524 | G/G | G/A | 0.068740 | 0.046388 | 0.011258 | 0.005037 | 0.000844 | 0.000750 | 0.001434 |
| rs2749612 | A/A | G/A | 0.109091 | 0.044380 | 0.007989 | 0.009080 | 0.002071 | 0.000681 | 0.000691 |
| rs335746 | C/C | A/A | 0.088328 | 0.052346 | 0.008008 | 0.003754 | 0.000748 | 0.000450 | 0.000387 |
| rs34471356 | A/A | A/T | 0.069825 | 0.037372 | 0.010578 | 0.005521 | 0.000575 | 0.000160 | 0.000459 |
| rs4333997 | C/C | T/C | 0.063936 | 0.048133 | 0.008849 | 0.005004 | 0.001148 | 0.000493 | 0.000471 |
| rs4350445 | G/G | G/T | 0.051887 | 0.030523 | 0.006385 | 0.002660 | 0.002887 | 0.000181 | 0.001182 |
| rs468696 | C/C | C/T | 0.077882 | 0.047981 | 0.007062 | 0.004933 | 0.001704 | 0.000510 | 0.000967 |
| rs6034343 | C/C | T/T | 0.067460 | 0.036459 | 0.022161 | 0.004754 | 0.000373 | 0.000621 | 0.000157 |
| rs7953166 | A/A | G/G | 0.042636 | 0.044452 | 0.012500 | 0.005721 | 0.000730 | 0.000105 | 0.000393 |
| rs842293 | G/G | G/T | 0.079589 | 0.047678 | 0.010406 | 0.004386 | 0.002161 | 0.001481 | 0.001387 |
| rs9564669 | C/C | C/T | 0.052590 | 0.047278 | 0.008914 | 0.004382 | 0.000766 | 0.000376 | 0.000146 |
| Detection ratio (Average) | - | - | 0.074748 | 0.046291 | 0.009777 | 0.005063 | 0.001016 | 0.000522 | 0.000538 |
| Result judgement | - | - | TURE | TURE | TURE | TURE | TURE | TURE | BIAS |

Table S5. Chimeric ratio detected in SNPs positions for each artificial chimera (the first test results of the second mixing experiment).

| Site | Receptor Genotypes | Donor Genotypes | Sample 1  (aCh1) | Sample 2  (aCh2) | Sample 4  (aCh4) | Sample 5  (aCh5) | Sample 6  (aCh6) | Sample 7  (aCh7) |
| --- | --- | --- | --- | --- | --- | --- | --- | --- |
| rs10140583 | G/G | A/A | 0.140832 | 0.065704 | 0.005003 | 0.000799 | 0.000585 | 0.000421 |
| rs10770083 | A/A | G/G | 0.153033 | 0.068241 | 0.004780 | 0.000802 | 0.000295 | 0.000153 |
| rs11076022 | A/A | G/G | 0.183116 | 0.076725 | 0.005967 | 0.000800 | 0.000662 | 0.000301 |
| rs11686988 | T/T | C/C | 0.142983 | 0.067767 | 0.005557 | 0.000555 | 0.000856 | 0.000322 |
| rs1290018 | C/C | C/T | 0.135639 | 0.056267 | 0.005729 | 0.000625 | 0.000306 | 0.000287 |
| rs1507072 | T/T | C/C | 0.135922 | 0.058641 | 0.004229 | 0.001151 | 0.000376 | 0.000443 |
| rs2211012 | G/G | T/T | 0.136333 | 0.063932 | 0.004915 | 0.000616 | 0.000316 | 0.000045 |
| rs2348478 | G/G | A/G | 0.149907 | 0.060070 | 0.007585 | 0.000186 | 0.000648 | 0.000880 |
| rs2469524 | G/G | G/A | 0.126547 | 0.059634 | 0.005785 | 0.000696 | 0.001123 | 0.000507 |
| rs2749612 | A/A | G/A | 0.145441 | 0.058184 | 0.005672 | 0.001396 | 0.001036 | 0.000868 |
| rs335746 | C/C | A/A | 0.135457 | 0.063476 | 0.004658 | 0.000690 | 0.000629 | 0.000069 |
| rs34471356 | A/A | A/T | 0.165040 | 0.076445 | 0.006658 | 0.000823 | 0.000648 | 0.000103 |
| rs4333997 | C/C | T/C | 0.124482 | 0.062636 | 0.005084 | 0.000540 | 0.000903 | 0.000651 |
| rs4350445 | G/G | G/T | 0.121614 | 0.054028 | 0.005740 | 0.001655 | 0.001010 | 0.000681 |
| rs468696 | C/C | C/T | 0.100171 | 0.045113 | 0.003512 | 0.000594 | 0.000360 | 0.000206 |
| rs6034343 | C/C | T/T | 0.095368 | 0.048077 | 0.004268 | 0.000640 | 0.000999 | 0.001178 |
| rs7953166 | A/A | G/G | 0.153273 | 0.071816 | 0.005029 | 0.000804 | 0.001105 | 0.000162 |
| rs842293 | G/G | G/T | 0.146253 | 0.065636 | 0.005400 | 0.000891 | 0.000932 | 0.000479 |
| rs9564669 | C/C | C/T | 0.142222 | 0.062441 | 0.005821 | 0.001012 | 0.000635 | 0.000447 |
| Detection ratio (Average) | - | - | 0.138612 | 0.062360 | 0.005336 | 0.000804 | 0.000707 | 0.000432 |
| Result judgement | - | - | TURE | TURE | TURE | TURE | TURE | BIAS |

Table S6. Chimeric ratio detected in SNPs positions for each artificial chimera (the second test results of the second mixing experiment).

| Site | Receptor Genotypes | Donor Genotypes | Sample 1  (aCh1) | Sample 2  (aCh2) | Sample 4  (aCh4) | Sample 5  (aCh5) | Sample 6  (aCh6) | Sample 7  (aCh7) |
| --- | --- | --- | --- | --- | --- | --- | --- | --- |
| rs10140583 | G/G | A/A | 0.145426 | 0.062541 | 0.004247 | 0.000589 | 0.000984 | 0.000480 |
| rs10770083 | A/A | G/G | 0.150039 | 0.067294 | 0.004911 | 0.000455 | 0.000380 | 0.000217 |
| rs11076022 | A/A | G/G | 0.147229 | 0.074266 | 0.006821 | 0.000531 | 0.000335 | 0.000181 |
| rs11686988 | T/T | C/C | 0.157328 | 0.062815 | 0.006150 | 0.000292 | 0.000483 | 0.000283 |
| rs1290018 | C/C | C/T | 0.133611 | 0.052285 | 0.004560 | 0.001165 | 0.000125 | 0.000279 |
| rs1507072 | T/T | C/C | 0.138385 | 0.068795 | 0.006328 | 0.000748 | 0.000515 | 0.000000 |
| rs2211012 | G/G | T/T | 0.153124 | 0.064529 | 0.004837 | 0.000457 | 0.000532 | 0.000000 |
| rs2348478 | G/G | A/G | 0.141102 | 0.061123 | 0.006557 | 0.001117 | 0.000282 | 0.001021 |
| rs2469524 | G/G | G/A | 0.121676 | 0.062649 | 0.005898 | 0.001268 | 0.000587 | 0.000487 |
| rs2749612 | A/A | G/A | 0.122006 | 0.055763 | 0.004984 | 0.000688 | 0.000743 | 0.001196 |
| rs335746 | C/C | A/A | 0.142943 | 0.054341 | 0.006128 | 0.000524 | 0.000493 | 0.000338 |
| rs34471356 | A/A | A/T | 0.162344 | 0.068182 | 0.006587 | 0.000386 | 0.000836 | 0.000000 |
| rs4333997 | C/C | T/C | 0.133893 | 0.057307 | 0.004129 | 0.000641 | 0.000416 | 0.000868 |
| rs4350445 | G/G | G/T | 0.115236 | 0.051895 | 0.005021 | 0.001278 | 0.000700 | 0.000999 |
| rs468696 | C/C | C/T | 0.107921 | 0.044992 | 0.004493 | 0.000344 | 0.000319 | 0.000381 |
| rs6034343 | C/C | T/T | 0.092507 | 0.053024 | 0.004102 | 0.000843 | 0.000953 | 0.000000 |
| rs7953166 | A/A | G/G | 0.146212 | 0.060416 | 0.004847 | 0.000410 | 0.000799 | 0.000000 |
| rs842293 | G/G | G/T | 0.135157 | 0.070574 | 0.006624 | 0.000961 | 0.001183 | 0.000600 |
| rs9564669 | C/C | C/T | 0.127418 | 0.056219 | 0.004669 | 0.000677 | 0.000553 | 0.000363 |
| Detection ratio (Average) | - | - | 0.135450 | 0.060474 | 0.005363 | 0.000704 | 0.000590 | 0.000405 |
| Result judgement | - | - | TURE | TURE | TURE | TURE | TURE | BIAS |

Table S7. Chimeric ratio detected in SNPs positions for each artificial chimera (the third test results of the second mixing experiment).

| Site | Receptor Genotypes | Donor Genotypes | Sample 1  (aCh1) | Sample 2  (aCh2) | Sample 4  (aCh4) | Sample 5  (aCh5) | Sample 6  (aCh6) | Sample 7  (aCh7) |
| --- | --- | --- | --- | --- | --- | --- | --- | --- |
| rs10140583 | G/G | A/A | 0.110816 | 0.063190 | 0.006155 | 0.000950 | 0.000970 | 0.000386 |
| rs10770083 | A/A | G/G | 0.125000 | 0.061830 | 0.005336 | 0.000227 | 0.000629 | 0.000218 |
| rs11076022 | A/A | G/G | 0.146161 | 0.061245 | 0.005318 | 0.000328 | 0.000294 | 0.000000 |
| rs11686988 | T/T | C/C | 0.130511 | 0.060388 | 0.005155 | 0.000395 | 0.000477 | 0.000000 |
| rs1290018 | C/C | C/T | 0.134629 | 0.062026 | 0.003985 | 0.000556 | 0.000586 | 0.000000 |
| rs1507072 | T/T | C/C | 0.152401 | 0.059839 | 0.006306 | 0.001549 | 0.000711 | 0.000231 |
| rs2211012 | G/G | T/T | 0.133397 | 0.061009 | 0.006206 | 0.000452 | 0.000424 | 0.000096 |
| rs2348478 | G/G | A/G | 0.116976 | 0.052574 | 0.005838 | 0.001210 | 0.001205 | 0.000520 |
| rs2469524 | G/G | G/A | 0.099840 | 0.057157 | 0.005564 | 0.000911 | 0.001066 | 0.000673 |
| rs2749612 | A/A | G/A | 0.152080 | 0.062786 | 0.003704 | 0.000389 | 0.000806 | 0.000234 |
| rs335746 | C/C | A/A | 0.120636 | 0.056956 | 0.005996 | 0.000288 | 0.000481 | 0.000000 |
| rs34471356 | A/A | A/T | 0.101449 | 0.049759 | 0.005064 | 0.000874 | 0.001143 | 0.000000 |
| rs4333997 | C/C | T/C | 0.136737 | 0.049973 | 0.006254 | 0.000825 | 0.001162 | 0.000979 |
| rs4350445 | G/G | G/T | 0.114244 | 0.065646 | 0.004887 | 0.001198 | 0.000799 | 0.000247 |
| rs468696 | C/C | C/T | 0.098854 | 0.052762 | 0.003694 | 0.000679 | 0.000533 | 0.000355 |
| rs6034343 | C/C | T/T | 0.126183 | 0.059524 | 0.003923 | 0.001516 | 0.000567 | 0.001163 |
| rs7953166 | A/A | G/G | 0.117714 | 0.061736 | 0.006348 | 0.000415 | 0.000651 | 0.000484 |
| rs842293 | G/G | G/T | 0.139513 | 0.071905 | 0.008405 | 0.000821 | 0.000802 | 0.001356 |
| rs9564669 | C/C | C/T | 0.121326 | 0.061446 | 0.005297 | 0.000799 | 0.000802 | 0.000140 |
| Detection ratio (Average) | - | - | 0.125183 | 0.059566 | 0.005444 | 0.000757 | 0.000743 | 0.000373 |
| Result judgement | - | - | TURE | TURE | TURE | TURE | TURE | BIAS |

Table S8. The informative STR loci and the results of multiple STR-PCR.

| Locus  designation | Chromosome location | Sample 1-1 (aCh1) | Sample 1-2 (aCh1) | Sample 1-3 (aCh1) | Sample 2-1 (aCh2) | Sample 2-2 (aCh2) | Sample 2-3 (aCh2) | Sample 3-1 (aCh3) | Sample 3-2 (aCh3) | Sample 3-3 (aCh3) |
| --- | --- | --- | --- | --- | --- | --- | --- | --- | --- | --- |
| D8S1179 | 8 | 0.116 | 0.138 | 0.109 | 0.078 | 0.081 | 0.070 | 0.028 | 0.022 | 0.056 |
| D21S11 | 21q11.2-q21 | 0.064 | 0.063 | 0.070 | 0.039 | 0.036 | 0.046 | 0.009 | 0.007 | 0.015 |
| CSF1PO | 5q33.3-34 | 0.060 | 0.064 | 0.063 | 0.038 | 0.027 | 0.037 | 0.004 | 0.004 | 0.007 |
| D3S1358 | 3p | 0.082 | 0.088 | 0.066 | 0.042 | 0.041 | 0.039 | 0.003 | 0.004 | 0.007 |
| TH01 | 11p15.5 | 0.141 | 0.151 | 0.108 | 0.084 | 0.089 | 0.076 | 0.051 | 0.032 | 0.025 |
| D3S317 | 13q22-31 | 0.104 | 0.109 | 0.123 | 0.088 | 0.085 | 0.076 | 0.042 | 0.046 | 0.045 |
| D16S539 | 16q24-qter | 0.104 | 0.128 | 0.122 | 0.045 | 0.068 | 0.072 | 0.007 | 0.008 | 0.017 |
| D2S1388 | 2q35-37.1 | 0.081 | 0.077 | 0.075 | 0.048 | 0.047 | 0.038 | 0.007 | 0.007 | 0.010 |
| TPOX | 2p23-2per | 0.111 | 0.154 | 0.119 | 0.070 | 0.132 | 0.106 | 0.026 | 0.051 | 0.037 |
| D18S51 | 18q21.3 | 0.119 | 0.107 | 0.108 | 0.054 | 0.067 | 0.058 | 0.005 | 0.007 | 0.008 |
| D5S818 | 5q21-31 | 0.111 | 0.104 | 0.099 | 0.072 | 0.079 | 0.031 | 0.039 | 0.039 | 0.034 |
| FGA | 4q28 | 0.121 | 0.119 | 0.136 | 0.123 | 0.099 | 0.085 | 0.094 | 0.072 | 0.096 |
| Detection ratio (Average) | - | 0.101 | 0.109 | 0.100 | 0.065 | 0.071 | 0.061 | 0.026 | 0.025 | 0.030 |

Continue

| Locus  designation | Chromosome location | Sample 4-1 (aCh1) | Sample 4-2 (aCh1) | Sample 4-3 (aCh1) | Sample 5-1 (aCh2) | Sample 5-2 (aCh2) | Sample 5-3 (aCh2) |
| --- | --- | --- | --- | --- | --- | --- | --- |
| D8S1179 | 8 | 0.171 | 0.179 | 0.147 | 0.112 | 0.128 | 0.100 |
| D21S11 | 21q11.2-q21 | 0.101 | 0.092 | 0.108 | 0.060 | 0.051 | 0.054 |
| CSF1PO | 5q33.3-34 | 0.092 | 0.084 | 0.094 | 0.040 | 0.043 | 0.041 |
| D3S1358 | 3p | 0.111 | 0.123 | 0.105 | 0.051 | 0.060 | 0.056 |
| TH01 | 11p15.5 | 0.168 | 0.172 | 0.152 | 0.093 | 0.112 | 0.089 |
| D3S317 | 13q22-31 | 0.142 | 0.140 | 0.153 | 0.105 | 0.090 | 0.090 |
| D16S539 | 16q24-qter | 0.154 | 0.162 | 0.166 | 0.080 | 0.092 | 0.072 |
| D2S1388 | 2q35-37.1 | 0.122 | 0.116 | 0.114 | 0.050 | 0.059 | 0.048 |
| TPOX | 2p23-2per | 0.205 | 0.198 | 0.178 | 0.135 | 0.151 | 0.086 |
| D18S51 | 18q21.3 | 0.177 | 0.164 | 0.146 | 0.078 | 0.075 | 0.078 |
| D5S818 | 5q21-31 | 0.129 | 0.158 | 0.160 | 0.084 | 0.084 | 0.082 |
| FGA | 4q28 | 0.177 | 0.156 | 0.189 | 0.140 | 0.128 | 0.132 |
| Detection ratio (Average) | - | 0.146 | 0.145 | 0.143 | 0.086 | 0.089 | 0.077 |

Table S9. XY-FISH results for each artificial mixture.

| Sample | Sample 1  (aCh1) | Sample 2  (aCh2) | Sample 3  (aCh3) | Sample 4  (aCh4) | Sample 5  (aCh5) | Sample 6  (aCh6) |
| --- | --- | --- | --- | --- | --- | --- |
| Detection number | 93/1000 | 56/1000 | 12/1000 | 6/1000 | 5/>1000 | 2/>1000 |
| Result judgement | TURE | TURE | TURE | TURE | FALSE | FALSE |
