# Supplementary figures and images for: The clinical application of SNP-based next-generation sequencing (SNP-NGS) for evaluation of chimerism and microchimerism after HLA-mismatched stem cell microtransplantation
Source: Int J Hematol. 2022 Jul 8;116(5):723–30. doi: 10.1007/s12185-022-03415-8 (PMC9588463; doi:10.1007/s12185-022-03415-8)

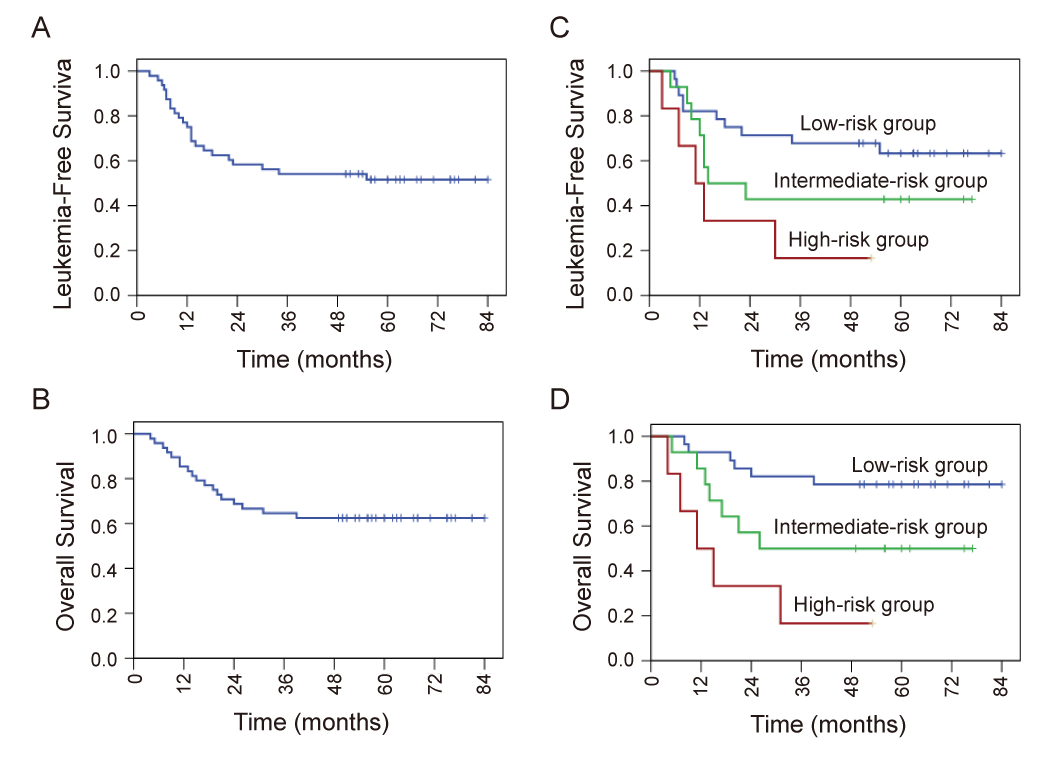

Supplement: Supplementary file 3 — Supplementary file3 (TIF 2722 kb) [file 12185_2022_3415_MOESM3_ESM.tif]
